# Characteristics of Otorhinolaryngological Emergencies in the Elderly

**DOI:** 10.4274/tao.2021.6193

**Published:** 2021-03-26

**Authors:** Sibel Yıldırım, Zahide Çiler Büyükatalay, Ahmed Majid Naji Agha Oghali, Rıdvan Kılıç, Gürsel Dursun

**Affiliations:** 1 Department of Otorhinolaryngology Head and Neck Surgery, Ankara University School of Medicine, Ankara, Turkey

**Keywords:** Emergency, geriatrics, maxillofacial injury, epistaxis, hospitalization, otorhinolaryngology

## Abstract

**Objective:**

This study was designed to characterize the distribution of otorhinolaryngological emergencies seen in the geriatric population in one year. In this article we present our results and discuss the differences between our results and those reported in the current literature.

**Methods:**

The study was carried out in a tertiary care university hospital. All patients aged 65 years or over that were referred by the general emergency department (ED) to the otorhinolaryngology emergency room in a one-year period were retrospectively reviewed. Demographic characteristics (age, gender), findings of physical examination, accompanying systemic diseases, diagnosis, and treatment methods were documented. Hospitalization and referral needs were also analyzed.

**Results:**

In the one-year period from April 2017 to April 2018, a total of 12,780 patients aged 65 or older presented to the ED and the otorhinolaryngology physician was consulted for 195 (1.5%) of these patients. The age range of the patients was 65–96 years, with a mean age of 75 years. The most common cause for presenting to the ED was maxillofacial trauma (31.7%), followed by epistaxis (18.7%). Dyspnea (9.7%) and peripheral facial paralysis (9.7%) were the third most frequent causes. The outcome analysis revealed that 9.7% of the patients were hospitalized.

**Conclusion:**

Identifying the characteristics of the geriatric patients presenting to EDs is important for developing proper management algorithms. Maxillofacial traumas were the most frequently seen ORL emergencies in our cohort of geriatric patients, followed by epistaxis. The distribution and the prevalence of the cases could differ according to the institutional protocols.

## Introduction

The geriatric population has become the fastest growing segment of the society as life expectancy increases worldwide. Compared to all other age groups, this population uses the most medical resources, has the longest hospital stays, as well as the highest rates of emergency department (ED) admissions (1). Overall, the elderly account for 15%–25% of ED visits (2).

These developments have inevitably increased the number of elderly patients seeking otorhinolaryngologic care. Otorhinolaryngology (ORL), as a specialty, deals with a wide range of health conditions, including communication disorders, allergies, and a variety of infections involving head and neck, as well as complex head and neck malignancies. Given this spectrum, one-third of the patients seen by the average otolaryngologist are over 65 years old (3). However, the principles of geriatric medicine and the issues specific to geriatric patients have not been sufficiently defined in relation to otorhinolaryngological emergencies.

Moreover, there are very few studies that have projected the epidemiological profile of geriatric otorhinolaryngological emergencies (1, 4). Therefore, this study was designed to determine the characteristics of the otorhinolaryngological emergencies seen in the geriatric population in one year in a single institution.

## Methods

The study was approved by the institutional ethics committee (number: 06-298-17) and conducted in accordance with the relevant privacy guidelines and applicable regulatory requirements. The data collected on the patients did not involve personal information or data that might affect patient privacy, and the study is exempt from the informed consent requirement due to its retrospective nature.

In this study, which was carried out at a tertiary care university hospital, we retrospectively reviewed the one-year data (2017-2018) involving all patients aged 65 years or over that were referred to the ORL emergency room from the general ED for consultation. Our hospital protocol for referrals states that patients do not have direct access to the ORL emergency room; instead, they need to be triaged by the ED physician to establish the severity of the emergency and the need for ORL evaluation.

Demographic characteristics (age, gender), findings of physical examination, accompanying systemic diseases, diagnosis, and treatment methods were documented, and the need for hospitalization and referral were analyzed. Descriptive statistical analysis was performed using Microsoft Excel (Microsoft Corp., Redmond, WA).

## Results

A total of 12,780 geriatric patients (aged 65 years or over) presented to the ED in the one-year period from April 2017 to April 2018. Of these patients, 195 (1.5%) were referred to the ORL physician for consultation. The age range of the patients was 65–96 years, with a mean of 75 years. Of these 195 patients 52.3% were in the youngest age group (65 to 74 years), 34.3% in the intermediate age group (75 to 84 years), and 13.3% were in the oldest age group (85+ years). The male to female ratio was 1.1:1. The mean age was 73 years (range: 65–96 years) for males and 76 years (range: 65–93 years) for females.

The most common cause for an ED visit was maxillofacial trauma (31.7%), followed by epistaxis (18.7%). Dyspnea (9.7%) and peripheral facial paralysis (9.7%) were the third most frequent causes (Table 1).

**Figure 1:**
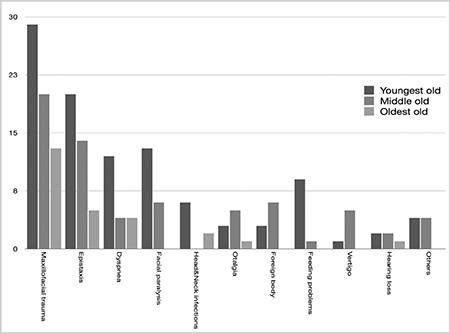
Distribution of cases by age groups

Analysis of the main complaints by age showed no significant differences. Maxillofacial traumas were the most common complaint, followed by epistaxis in all age groups. Their distribution among the age groups, however, were different. Maxillofacial traumas constituted 50% of the diagnoses in the eldest group, 29.8% in the intermediate age group, and 28.4% in the youngest age group. The third most common complaint was peripheral facial paralysis in the youngest and intermediate age groups. On the contrary, dyspnea was the third most common complaint in the eldest age group. The distribution of the diagnoses by age groups is shown in Figure 1.

Of the maxillofacial traumas, 79% (n=49) were caused by falls, 17.7% (n=11) were associated with motor vehicle accidents, and 3.2% (n=2) were assault related. There were 59.6% (n=37) simple traumas (such as skin laceration, incision, bleeding, hematoma) not accompanied by a fracture; and 40.3% (n=25) had fractures in one or more of the maxillofacial bones. Simple traumas were managed with wound care, dressing, laceration repair, and medical treatments (analgesic, antibiotics) in the ED. Traumas with fractures were evaluated for any functional or cosmetic deformities, and reduction was planned for displaced fractures. Six patients with displaced nasal fracture underwent closed nasal bone reduction under local anesthesia in the ED and four patients refused reduction. Patients with mandible, skull base, and maxilla fractures needed hospitalization, and interventions were planned in the operating room under general anesthesia. Patients with non-displaced fractures did not require any intervention. The distribution of the fractures is listed in Table 2.

The second most encountered emergency was epistaxis, accounting for 18.7% of all cases. Hypertension was the most frequent accompanying systemic disease, with a rate of 40.5%, followed by cardiac pathologies (atrial fibrillation, valve diseases) (32.4%). In 27% of the patients, there were no known systemic diseases. The use of anticoagulant medication was reported by 51.5% of the patients. Treatment modalities used for the management of epistaxis are shown in Table 3. Only one patient was hospitalized with posterior nasal packing.

Cases with dyspnea ranked third in prevalence (9.7%). Obstruction of the tracheostomy cannula with a crust or mucous plug was the most common cause of dyspnea (31.5%). These patients were discharged after tracheobronchial suctioning and removal of stiff plugs. Other causes of dyspnea and the treatment modalities are listed in Table 4. Hospitalization rate was the highest (63%) for dyspnea cases compared to the other emergencies.

Peripheral facial paralysis also accounted for a large number (9.7%) of the emergencies, all of which were evaluated as idiopathic. Corticosteroid treatment were planned, and doses were adjusted according to patients’ comorbidities, such as hypertension and diabetes mellitus. Only one patient needed hospitalization because of uncontrolled hypertension.

Head and neck infections accounted for 5.1% of the emergencies. In 40% of these cases, infection had progressed to deep neck space abscesses. These cases all required drainage except for one that had a retropharyngeal abscess. This patient was hospitalized and monitored for regression under intravenous antibiotherapy.

Otalgia was the main complaint of 10 patients, with the most common etiology being acute/chronic otitis media, followed by auricular abscess. A foreign body was a relatively common emergency and mainly located in the larynx (33.3%). Feeding problems were another reason for ORL consultation and mainly secondary to head and neck malignancies (55.5%).

Seven patients diagnosed with dizziness or vestibular impairment were referred for ORL consultation. Of these cases, two with neurological deficits were referred to department of neurology. Two patients showed no vestibular deficit in vestibular examinations. Hearing loss was a less common complaint for ED admission, accounting for 2.5% of the cases. Only one patient had a true emergency with sudden sensorineural hearing loss; the other cases were related to earwax or presbycusis.

The outcome analysis revealed that 9.7% of the patients required hospitalization. Distribution of the hospitalization rates by diagnoses is shown in Table 5.

## Discussion

The geriatric population is the fastest growing segment of society. It is estimated that 12.5% of the world’s population will be aged over 65 years by 2030, and all physicians will be required to deal with the effects of this aging population (5). Accordingly, it is reasonable to expect an increase in the number of elderly patients who need emergency otorhinolaryngological care. Extended reviews of ORL emergencies have been conducted before; however, the profile of ORL emergencies in the geriatric population has not been adequately addressed (6, 7, 8, 9). Therefore, this study provided a comprehensive analysis of 195 geriatric patients referred to ORL from an ED.

In the presented study, the referral rate of geriatric patients to the ORL physician was found as 1.5%. In our institution, patients do not have direct access to an ORL physician; all patients are first examined and treated by an ED physician. Therefore, this rate reflects not the prevalence of geriatric ORL emergencies, but the geriatric emergency patients referred for evaluation through the triage system. It is difficult to determine the real prevalence of geriatric ORL emergencies since each institution has a different patient flow system, and management and referral protocols can vary according to each hospital’s management protocol and the clinicians’ approaches.

Maxillofacial traumas were the predominant reason for referral in our cohort. This is in contrast to the study of Dagan et al. (1), in which the most common reason for ORL ED admission among geriatric patients was balance disorders. This may be explained by the different management protocols of the respective institutions. Our study was conducted at a university hospital with a trauma center. Therefore, most of the trauma emergency calls in the city were directed to our hospital, which led to an increased rate of trauma cases. Furthermore, at our institution, the plastic surgery department is situated on another campus, meaning most maxillofacial trauma cases are generally referred to ORL, which also led to increased rates. In our study, most of the traumas were due to falls and more common in the eldest group. The physical changes in the elderly, such as muscle weakness, low levels of physical activity, balance and gait disorders, and visual impairment, could explain this result. The nasal bone was the most common fracture site, followed by the maxilla. Falcone et al. (10) stated similar results but there are different studies those mandible fractures preponderant (11, 12). Reduction was offered for all the displaced fractures, but 28.5% of the patients refused treatment. It is not possible to compare this rate with that of other age groups since there are no data on the treatment refusals in maxillofacial fractures. However, we deem that refusal rates are higher in the geriatric population due to less concerns about cosmetic deformity.

Epistaxis, which was the second most encountered emergency, can be challenging in the elderly, especially because their use of multiple medications and the presence of systemic disease comorbidities compound the difficulties in their treatment. In their analysis of the epidemiology of ORL emergencies, Lammens et al. (6) reported epistaxis as the most frequent diagnosis, accounting for about 25% of their cases. In our study, epistaxis accounted for 18.7% of the patients, which is slightly lower than the percentage reported by the referred study. This difference can be explained by the study design. In the referred study, all epistaxis cases were assessed under the heading of epistaxis, whereas in our study traumatic epistaxis was included in the maxillofacial trauma group. Prompt management of epistaxis is important for hemodynamic stability, especially in the elderly (13). None of the patients in our study required resuscitation or intensive care unit follow-up. In general, their epistaxis cases were not severe, and anterior nasal packing were sufficient. Only one patient had posterior-based epistaxis that required posterior nasal packing and hospitalization for observation.

Among the most frequent complaints, dyspnea was the third in our study, a finding which differs from other epidemiological studies (7, 8, 14). We assume that the difference is due to our study’s geriatric age group in which respiratory distress occurs more frequently than in younger age groups (15). Dyspnea in the elderly is often the consequence of multiple overlapping disorders, but in our study, only the dyspnea cases involving upper airway pathologies were evaluated. The most common etiologies were mainly associated with head and neck malignancies, which are also more commonly seen in this age group (16). Hospitalization need was significantly higher in dyspnea cases than for other emergency cases because airway pathologies need close observation during medical or surgical treatments.

Other causes that are listed in the literature for ORL referrals in the elderly are peripheral facial paralysis, head and neck infections, otalgia, foreign body, feeding problems, balance disorders, and hearing loss. Almost all commonly seen ORL conditions were presented in our results, but the rate of vertigo was found remarkably lower compared to the reports in the literature. This can be explained by the differences in patient admission protocols. In our institution, all patients admitted to the EDs are triaged by emergency medicine specialists, and the decision of whether the patient should be referred to the neurologist or the otorhinolaryngologist is based on the ED physician’s clinical judgement. The general approach of the ED physicians is to rule out a neurological emergency, so they usually first refer patients to the neurologist. In the absence of a central pathology, patients are evaluated for peripheral vertigo, in which case, most of the time, symptomatic treatment is applied and ORL outpatient follow-up is recommended. With this algorithm, ORL generally evaluates vertigo patients in outpatient clinics rather than in the emergency unit.

The retrospective design of the study did not allow us to comment on the suitability of the referrals; however, review of the cases showed that most were genuine emergencies. Gallo et al. (17) found that the vast majority of ORL ED admissions were not real emergencies, but their ED working protocol is different from the one at our institution. In the referred study, patients had direct access to ORL ED without any triage system. In our ED, however, all patients are first seen by an ED physician in the triage room, and non-emergency cases are redirected. That the need for ORL consultation is determined by the ED physician, helps reduce unnecessary ORL admissions.

## Conclusion

Maxillofacial traumas are the most frequently seen ORL emergencies among geriatric patients, followed by epistaxis. Higher hospitalization rates are seen in dyspnea. The elderly population is physiologically different from younger adults, and they have different needs. Identifying the characteristics of geriatric patients presenting to the ED is important for developing proper management algorithms. Multicenter studies with larger cohorts will better define the epidemiological profile of the geriatric ORL emergencies.

**Main Points**

• Identifying the characteristics of geriatric patients presenting to the ED is important for developing proper management algorithms.

• In geriatric patients, the most frequently seen ORL emergencies are maxillofacial traumas, followed by epistaxis.

• Higher hospitalization rates were seen in patients with dyspnea.

• The distribution and the prevalence of the cases could differ according to the institutional protocols.

## Figures and Tables

**Table 1:**
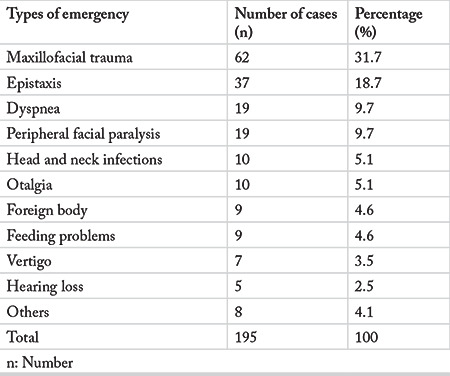
Distribution of patients by type of emergency disorders

**Table 2:**
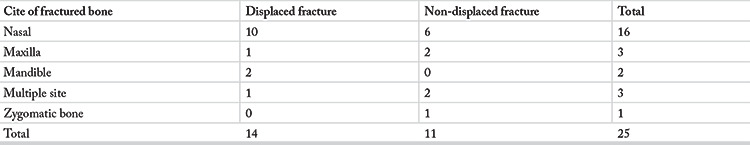
Numbers of fractures by site and displacement

**Table 3:**
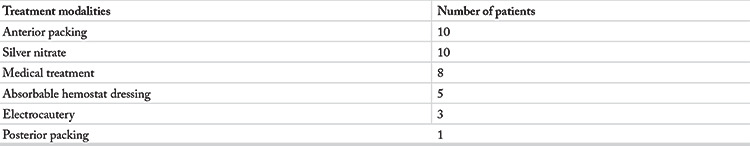
Treatment modalities for epistaxis

**Table 4:**
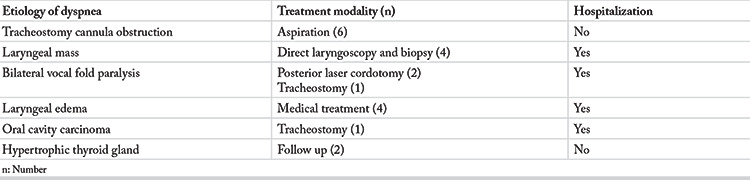
Dyspnea cases according to the etiology, treatment modality and hospitalization need

**Table 5:**
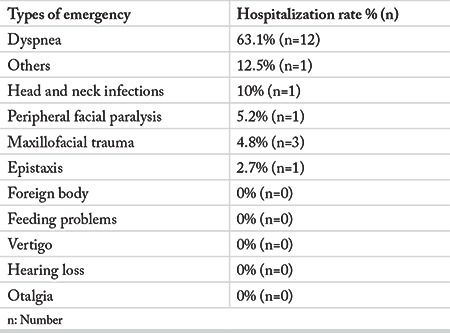
Hospitalizations by diagnoses
